# Reviewed article: Zanlourensi CB, Boing AF. Intrastate inequality of COVID-19 vaccination coverage: spatial analysis and socioeconomic status in Santa Catarina, Brazil (2021-2023). Epidemiol Serv Saude. 2025:34;e20240329

**DOI:** 10.1590/S2237-96222025v34e20240329.a

**Published:** 2025-08-08

**Authors:** Leonardo Trench

**Affiliations:** USP FOB, Saúde Coletiva

**Table:** 

Rating	Excellent	Good	Average	Below average	Poor
Interest	✓				
Quality				✓	
Originality	✓				
Overall		✓			

**Table:** 

Questionnaire	Yes	No	Not applicable
Does the manuscript contain new and significant information to justify publication?	✓		
Does the Abstract (Summary) clearly and accurately describe the content of the article?		✓	
Is the problem significant and concisely stated?	✓		
Are the methods described comprehensively?	✓		
Are the interpretations and conclusions justified by the results?	✓		
Is adequate reference made to other work in the field?	✓		

## Manuscript Structure

Length of article is: Too long

Number of tables is: Adequate

Number of figures is: Excessive

Please state any conflict(s) of interest that you have in relation to the review of this paper (state “none” if this is not applicable): None

## Comments

Vejo que a discussão traz na verdade relatos dos resultados ou invés de discuti-los, ou comparações com outras regiões de maneira muito genérica, o que diminui a relevância do mesmo quanto a tomada de decisões, como por exemplo mudanças nas formas de comunicação, para a região ou que possa se extrapolar para geral, que creio seria um dos grandes ganhos sociais deste trabalho.

Há uma quantidade excessiva de gráficos e mapas, o que dificulta a leitura e pode levar a perda o interesse por parte do leitor, talvez se reduzisse para os que apresentam os dados mais interessantes, mais relevantes para o que se deseja mostrar. Esse tipo de mapa, que traz os municípios ele faz um mosaico que fica cansativo se não estiver bem explicado no texto e na legenda. Entendo que o objetivo são os municípios, mas acho importante fazer um link muito bem feito para não tirar o interesse do leitor de algo que pode ser muito importante para nortear, por exemplo, as ações em comunicação em saúde.

Os gráficos, por exemplo, todos mostram o mesmo desenvolvimento do fato para todas as faixas etárias, então será que não seria possível consolidá-los em menos gráficos?

Se o objetivo é analisar os dados, precisamos avaliá-los de maneira a trazer na discussão informações que possam ser úteis, e dada a urgência e constância das constatações, um modelo de tradução da evidência seria bastante interessante.

Considero o delineamento do trabalho satisfatório para o objetivo, a descrição que deixa um pouco a desejar. Como são utilizados métodos menos corriqueiros, acho importante descrever corretamente os conceitos dos termos utilizados.

Quanto às siglas, importante lembrar que depois que aparece a sigla, não precisamos usar mais o termo completo, e isso ocorre várias vezes com a cobertura vacial, por exemplo.

Seguem algumas informações detalhadas sobre linhas e páginas:

Página 3 a partir da linha 48.

Como o Índice de Moran Local Univariado não é um índice usualmente utilizado, seria importante ao descrever os clusters como alto-alto, baixo-baixo, alto-baixo e baixo alto(há um erro de digitação na linha 55), seria importante descrever os parâmetros que norteiam essa classificação.

Pag 4 linha 10:

Esses resultados não estão nas tabelas ou figuras, seria interessante ter esse consolidado.

Como não são todas as pessoas que conhecem as peculiaridades geográficas do estado, acho interessante ter um mapa com as regiões do estado, para que possamos correlacionais os mapas com o que está escrito.

Dentro das tabelas podemos simplificar as visualizações. Desnecessário usar tantas vezes os termos “anos”, uma vez que pode ficar explicitado isso logo na primeira linha do item.

Na tabela 1, importante explicar corretamente o que são os quintis, já que é uma medida não corriqueira e precisa ficar melhor explicitada dentro do contexto do trabalho.

